# Biomedical effects of *Laurus nobilis L*. leaf extract on vital organs in streptozotocin-induced diabetic rats: Experimental research

**DOI:** 10.1016/j.amsu.2020.11.051

**Published:** 2020-11-21

**Authors:** Rebin Rafaat Mohammed, Abdullah Khalid Omer, Zabit Yener, Ahmet Uyar, Avin Kawa Ahmed

**Affiliations:** a Sulaimani Veterinary Directorate, Chamchamal Veterinary Hospital, Sulaimani, Iraq; b Department of Food Hygiene and Quality Control, Faculty of Veterinary Medicine, Urmia University, Urmia, Iran; c Department of Pathology, Faculty of Veterinary Medicine, Van Yuzuncu Yil University, Van, Turkey; d Department of Pathology, Faculty of Veterinary Medicine, Hatay Mustafa Kemal University, Hatay, Turkey

**Keywords:** Diabetes mellitus, *Laurus nobilis*, Streptozotocin, Histopathology, Blood glucose

## Abstract

Diabetes mellitus (DM) has been treated with herbs for centuries and many herbs reported to exert antidiabetic activity. *Laurus nobilis* is an aromatic herb belonging to the *Lauraceae* family, commonly known as bay. This study aimed to investigate the activity of *Laurus nobilis* leave extracts on histopathological and biochemical changes in β-cells of streptozotocin (STZ)-induced diabetic rats. Thirty healthy adult male albino rats were included in the study and divided equally into 5 groups for 4 weeks as follow; control group (C), diabetic group (D), diabetic *Laurus nobilis* extract group (DLN), *Laurus nobilis* extract group (LN) and diabetic acarbose (DA) group. Histopathologically, D group rats exhibited various degenerative and necrotic changes in their liver, pancreas and kidney, whereas the DLN rats had nearly normal histology. Insulin immunostaining in the pancreatic beta cells was decreased in the D group compared to the C group, whereas the DLN group was similar to the C group. The glucose concentration decreased significantly in both diabetic rats treated with *L. nobilis* and acarbose (*p* < 0.05). Additionally, the levels of aspartate aminotransferase (AST), gamma-glutamyltransferase (GGT) and alanine aminotransferase (ALT) enzyme were significantly decreased in both diabetic rats treated with *L. nobilis* and acarbose, compared to the D group (*p* ˃ 0.05). Outcomes of this study said that leave extracts of *L. nobilis* has valuable effect on blood glucose level and ameliorative effect on regeneration of pancreatic islets, it also restored the altered liver enzymes, urea, creatine kinase, total protein levels, calcium and ferritin to near normal.

## Highlights

• Diabetes mellitus has been treated with herbs for centuries and many herbs reported to exert antidiabetic activity.
• Historically, traditional herbal treatments have been shown to possess successful pharmacological activity, such as in the case with metformin, isolated from Galega officinalis.
• *Laurus nobilis* has a valuable effect on blood glucose level and ameliorative effect on regeneration of pancreatic islets.
• *Laurus nobilis* have also been shown to display insulin-enhancing activity in vitro.
• *Laurus nobilis* also restored the altered liver enzymes, urea, creatine kinase, total protein levels, calcium and ferritin to near normal.

## 1 Introduction

Diabetes mellitus (DM) is a chronic endocrine disorder of multiple etiologies distinguished by hyperglycemia resulting from defects in insulin secretion, insulin action, or both [1]. The clinical diagnosis of diabetes is often indicated by the presence of symptoms such as polyuria, polydipsia, unexplained weight loss, and is confirmed by documented hyperglycemia [2,3]. Diabetes complications can be classified as microvascular complications such as nervous system damage (neuropathy), renal system damage (nephropathy) and eye damage (retinopathy), and macrovascular complications for example cardiovascular disease, stroke, and peripheral vascular disease [[4], [5], [6]].

Natural supplements are widely used around the world to treat diabetes, but medical research does not support their effectiveness. DM has been treated with herbs for centuries and there are many herbs that have been reported to exert antidiabetic activity [[7], [8], [9]]. Traditional plants have been used as a cure for diabetes for a long time before the introduction of modern medicine. Historically, traditional herbal treatments have been shown to possess successful pharmacological activity, such as in the case with metformin, isolated from Galega officinalis [[10], [11], [12]].

The dried leaves of *Laurus nobilis* are used as a spice or flavoring agent in the culinary and food industries [13]. The essential oil (EO) prepared from the leaves has been reported to have antibacterial, antioxidant and anti-inflammatory activities [14,15]. Further leaves of *Laurus nobilis* have been also used to treat rheumatism, neuralgia, and scabies [16,17].

The main volatile compounds in laurel herb extract are usually 1,8-cineole, methyl eugenol, α-terpinyl acetate. α-pinene, β-pinene, sabinene, and linalool. Generally, leaves and berries are widely utilized, oxygenated monoterpene 1,8-cineole is one of the major constituents of leaves and berry fruits essential oil obtained from *Laurus nobilis* [18]. The leaves contains about 1.3% essential oils. The EOs obtained of berry fruit depending on provenance and storage conditions. The oil extracted from berries contain fatty acids, which include lauric (54%) linoleic (17%) oleic (15%) and palmitic (5%) and volatile compounds such as β-ocimene (22%), 1,8-cineole (9.5%), bicyclogermacrene (4.5%) and β-elemene (2%) [19,20]. The bioactive components in bay leaves have been shown to have effects on insulin sensitivity, glucose uptake, antioxidant status, inflammatory response, and glucose emptying.

*Laurus nobilis* is an aromatic herb belonging to the *Lauraceae* family known as bay, daphne, bay laurel, true bay, or sweet bay. It is an evergreen tree or shrub, is a native plant from the Southern Mediterranean region, found in warm climate regions with high rainfall, especially distribution in Turkey, Greece, Spain, Portugal, Italy, France, Morocco and Mexico [21,22]. In Turkey, *Laurus nobilis* grows in the Marmara, Aegean and Mediterranean regions [23,24]. It's one of the oldest known spices, widely used as a condiment and spice. With relevant medicinal properties due to its important chemical composition and its potential therapeutic effects [14]. Afifi et al. [25] reported that aqueous extracts of *Laurus nobilis* seeds were effective in reducing ethanol-induced gastric ulcer in rats.

Traditionally bay leave extract have been used orally to treat the symptoms of gastrointestinal problems, such as epigastric bloating, impaired digestion, eructation, and flatulence [25]. As a traditional medicine, the decoction or tea of bay leaves is often used as therapy, intestinal and gastric antispasmodic, against diarrhea, for rheumatic pains, also diseases of the respiratory tract, as a cough, asthma, and cardiac diseases [26].

Bay leaves have also shown that display insulin-enhancing activity in vitro [27], however these enhance glucose metabolism and the overall condition of individuals with diabetes not only by hypoglycemic effects but also by improving lipid metabolism, antioxidant status, and capillary function [28]. Bay leaves reduced serum glucose, total cholesterol, LDL cholesterol and triglycerides, and increased HDL-cholesterol levels in people with type 2 diabetes [27]. This study aimed to evaluate the effects of *Laurus nobilis* leave extracts on biochemical and histopathological changes of pancreas, liver and kidney on diabetic rats.

## 2 Materials and methods

### 2.1 Materials

#### 2.1.1 Animals

All experimental protocols were approved by the Experimental Animal Center of Yuzuncu Yil University, Van, Turkey. In the present study, thirty male Wistar rats, weighting about 160–200 g with averagely 7 weeks old were randomly divided into five groups based on treatment each group containing 6 animals. All animals were housed under safe laboratory conditions in a temperature-controlled room (22–24 ^°^C) and kept on a 12 h light/dark cycle. Blood glucose and body weight were monitored before treatment once a week throughout 4 weeks of experimental period.

#### 2.1.2 Equipments

All measurements were performed by using Automatic tissue processor (LEICA TP 1020 Semi-enclosed Benchtop), Centrifuge (Hettich TD4, Shanghai, China), Nikon digital camera (DXM-1200F), Glucometer (Accu-Chek, Taiwan), Tissue imbedded paraffin (LEICA Eg115° H Shanghai, China).

### 2.2 Methods

#### 2.2.1 Preparation of plant material and diet

The bay leave purchased from traditional herbal markets, a specimen was deposited at the herbarium of the Hatay. The *laurus nobilis* leaves were ground to powder by electric grinder and extracted with ethanol, and the extraction oils were stored at room temperature in dark place. 200 mg kg ^−1^ of bay oils was administered daily orally using intragastric tube at the time of work [29].

#### 2.2.2 Diabetes model with streptozotocin (STZ)

Diabetes mellitus was induced by single intraperitoneal (IP) injection of freshly prepared STZ (Sigma-aldrich, Saint Louis, MO) at dose of 70 mg kg ^−1^ b.w. dissolved in 0.01 M citrate buffer, pH 4.5 [30]. After 72 h of STZ injection, and overnight fast, blood was taken from tail artery of the rats. Accu-Chek monitoring used to rapidly changing blood glucose level, when rats with blood glucose higher than 250 mg dl ^−1^ were selected for the diabetic groups and involved to the examination. Strict monitoring of all diabetic group rats was done for blood glucose after 24–48 h of STZ administration.

Injection of STZ and attack on pancreas cause hypersecretion of insulin and this lead to intensive hypoglycemia and this may cause death to many animals, to avoid this, drinking water containing 10% dextrose were given to rats directly after I.P of STZ. In addition for taken care about rats, blood glucose was measured at 3rd, 15th and 28th days of throughout experimental model in blood taken from tail artery.

#### 2.2.3 Experimental protocols

Experimental animals were randomly divided into 5 groups; each group was included 6 animals. The examination period was continuous for four weeks as below:

1. Control group (C): did not receive any other kind of co-supplementation. Rats were given a standard diet.
2. Diabetes group (D): in this group diabetes was induced by administered 70 ml/kg single dose of STZ IP injection [30], and given standard diet.
3. Diabetes treated with *Laurus nobilis* leave extract group (DLN) given 200 mg kg ^−1^ of bay extract that administered every day orally using intragastric tube for 28 days during the examination [29].
4. *Laurus nobilis* extract group (LN): 200 mg kg ^−1^ of bay leave was administered every day orally using intragastric tube [29].
5. Diabetes with drug (Acarbose) group (DA): The rats of this group were treated with 150 mg kg ^−1^ dose of Acarbose tablet (Glucobay), (Bayer Türk Kimya San) each day orally using intragastric tube [31].

#### 2.2.4 Blood sample collection and biochemical analysis

At the end of the treatment period, all rats were fasted for 18 h, weighed and then anaesthetized via IP injection of ketamine hydrochloride (50 mg kg ^−1^ b.w.) and xylazine (8 mg kg ^−1^ b.w.). Blood samples were collected from the heart puncture of rats and transferred to suitable tubes for biochemical analysis using Merck commercial diagnostic kits (Darmstadt, Germany) on a blood chemistry analyzer (BTS-350, BioSystems S.A. Barcelona, Spain).

#### 2.2.5 Histopathological studies

Histopathological investigation were performed at last day of the experiment. Pancreas, liver and kidneys were removed and kept in 10% formaldehyde and embedded in paraffin. Blocks of the preserved tissues were sectioned (3–5 μm) on a microtome (Leica RM 2135: Leica Biosystems Nussloch GmbH, Nussloch, Germany) and mounted on glass slides. Hematoxylin and eosin (H&E) staining was done and histopathological examination was done in accordance with the method adopted by Nagy and Ewais [32].

#### 2.2.6 Immunohistochemical investigation

Insulin expressions were stained using the streptavidin-peroxidase method (ABC), with the streptavidin / biotin immunoperoxidase kit (Histostain-Plus Bulk Kit; Zymed, South San Francisco, CA, USA) in accordance with the staining procedures of the manufacturer companies. After the sections taken in 4–5 micron thick by microtome had been placed on adhesive slides, they were passed through xylene and alcohol series. In order to remove the endogenous peroxidase activity, the sections were kept in 3% Hydrogen peroxide (H_2_O_2_) for 20 minutes after being washed with PBS (phosphate buffer solution). After placing the antigen in the retrieval solution (citrate buffer), it was heated twice in the microwave oven for 20 minutes. Then it was taken out of the oven and it was left to cool until it reached room temperature. After being washed again with PBS, the sections were blocked by protein blocking (non-immune serum) for 20 minutes. Insulin antibody (Catalog no: ab-181547; Abcam, Toronto, Canada, diluted 1:1.000) were dropped into each tissue and left overnight at +4°C. Sections were washed again with PBS and incubated for 20 minutes at room temperature with biotinylated secondary antibody. The sections washed again with PBS were left in streptavidin-peroxidase for 20 minutes and then washed in the same way as PBS. After washing, 3,3'-Diaminobenzidine (DAB) was dropped and left for 1–2 minutes. Then all sections were kept in Mayer’s hematoxylin (Bio-Optika, 05-06002E) for 1–2 minutes and washed in tap water. Sections were passed through of 70%, 80%, 90%, 96% alcohol for 3 minutes, respectively, and 100% alcohol for 10 minutes and of the xylol series for 5 minutes were closed using entellan. Negative controls reacted with PBS were used instead of primary antibodies to confirm staining. Sections were examined and photographed under a light microscope. The immunoreactivity staining intensities of the testicular samples obtained from the groups with primary antibodies were scored as mild (+), moderate (++) and strong (+++).

#### 2.2.7 Statistical analysis

Statistical analyses were performed by using ‘IBM SPSS Alan C. Elliott software. ANOVA test was applied to analysis the significant differences in all groups. (Kruskal-Wallis) huc Dunn's test was done to compare among groups, differences were considered significant when the *p* < 0.05.

## 3 Results

### 3.1 Effect on body weight

The diabetic rats exhibited profound body weight loss as compared to normal rats. The initial, half-way and final body weights of the rats are shown in (Table 1).

**Table 1:** Results of body weight (g) in different groups (mean ± Standard deviation).

G	1st day	15th day	28th day
C	195.33 ± 15.31^a^	233.66 ± 17.90 ^b^	268.00 ± 13.91 ^b^
D	204.33 ± 19.28^b^	195.83 ± 33.18^a,b^	189.66 ± 24.76^a^
DLN	217.33 ± 19.00^a^	211.66 ± 10.38^a^	214.33 ± 10.46^a^
LN	212.33 ± 13.10^a^	229.83 ± 17.26^b^	262.16 ± 21.16^b^
DA	215.66 ± 18.85^a^	213.83 ± 24.11^a,b^	211.16 ± 34.56^a^

*G; groups were C (control group), D (diabetic group), DLN (diabetic with *Laurus nobilis* trated group), DA (diabetic with drug treated group), LN (*Laurus nobilis* fed group).

Data are means ± S.D. ^b,c,d^Significantly different from Control group in initial.

The final body weights in diabetic group (D), diabetes *Laurus nobilis* group (DLN), and diabetic Acarbose group (DA) were significantly decreased when they compared with measuring at 14 days and also higher decreased compared with baseline weight at the beginning of the study. In contrast, body weights in control group (C) were significantly higher in comparison to other groups during experimental period while *laurus nobilis* group (LN) was not significantly changed in body weight. Diabetes seemed to be more effective in decreasing body weight (Table 1).

### 3.2 Effect on serum glucose

The serum glucose concentration (mg dl ^−1^) in the Group C was significantly (*p* < 0.05) lower, whereas Group D showed significantly (*p* < 0.05) higher concentration as compared to other groups throughout the experiment (Table 2). On the other hand, *L*. *nobilis*-fed group rats showed relative to control (C) rats during the first, second and fourth week, respectively.

**Table 2:** Results of blood glucose (mg dl ^−1^) in different groups (mean ± Standard deviation).

G	1st day	15th day	28th day
C	128.16 ± 10.87^a^	157.50 ± 30.73^a^	138.33 ± 24.23^a^
D	600.00 ± 0.00 ^d^	592.50 ± 18.37^c^	457.66 ± 170.57^c^
DLN	563.16 ± 53.21^c^	479.16 ± 164.36 ^b^	287.33 ± 109.83 ^b^
LN	120.00 ± 4.97^a^	127.33 ± 20.61^a^	140.00 ± 7.29^a^
DA	521.66 ± 14.26 ^b^	587.33 ± 18.61^c^	316.16 ± 70.00 ^b^

*G; groups were C (control group), D (diabetic group), DLN (diabetic with *Laurus nobilis* trated group), DA (diabetic with drug treated group), LN (*Laurus nobilis* fed group).

Data are means ± S.D. ^b,c,d^Significantly different from Control group.

The two diabetic treated Groups (DLN and DA) showed significant (*p* < 0.05) decrease in glucose concentration on last week and as compared to Group C; the decrease was the highest in Groups C and LN. All Higher blood glucose concentrations were observed in group D, DLN and DA group after 72 h of STZ injection, and gradually decreased on day 14th and 28th also significant difference between group D and treated diabetes groups (DLN, DA). *Laurus nobilis* inhibited the development of diabetes induced by STZ treatment (Table 2).

### 3.3 Histopathological findings

#### 3.3.1 Liver

**Fig. 1:**
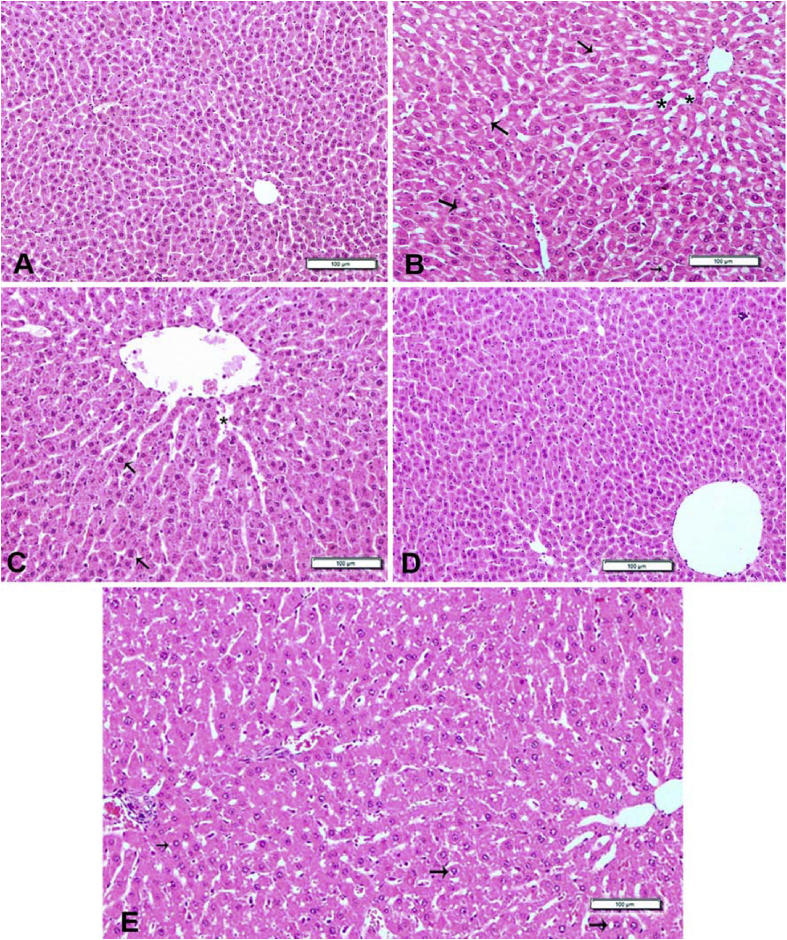
Hematoxylin and eosin-stained sections of liver. **A)** Control group: Normal histological appearance. **B)** Diabetic group: Disseminated vacuolization (arrows) in the hepatocytes and dilation of sinusoids (stars). **C)** Diabetic + *L. nobilis* treated group: Slight hydropic degeneration in the some hepatocytes (arrows) and dilation of sinusoids (star). **D)***L. nobilis*-fed group: Almost normal histological appearance of the liver. **E)** Diabetic + drug- treated group: rat administered with acarbose showing slight hydropic degeneration (arrows) and dilation of sinusoids. Bar = 100 μm.

The normal histological view of the liver was observed in the Control and LN groups (Fig. 1-A and D). Degeneration and necrosis in the hepatocytes were detected in Diabetes group. Additionally, disrupted hepatic cords and sinusoidal architecture were detected. Varying in size vacuoles were determined in the cytoplasm of degenerated hepatocytes (Fig. 1-B). These findings were found to be significantly reduced in the liver of rats in DLN group such as degeneration and necrosis (Fig. 1-C). Similar histological appearance to the Control group were found in DA group except for slight hydropic degeneration and dilation of sinusoids (Fig. 1-E).

#### 3.3.2 Pancreas

**Fig. 2:**
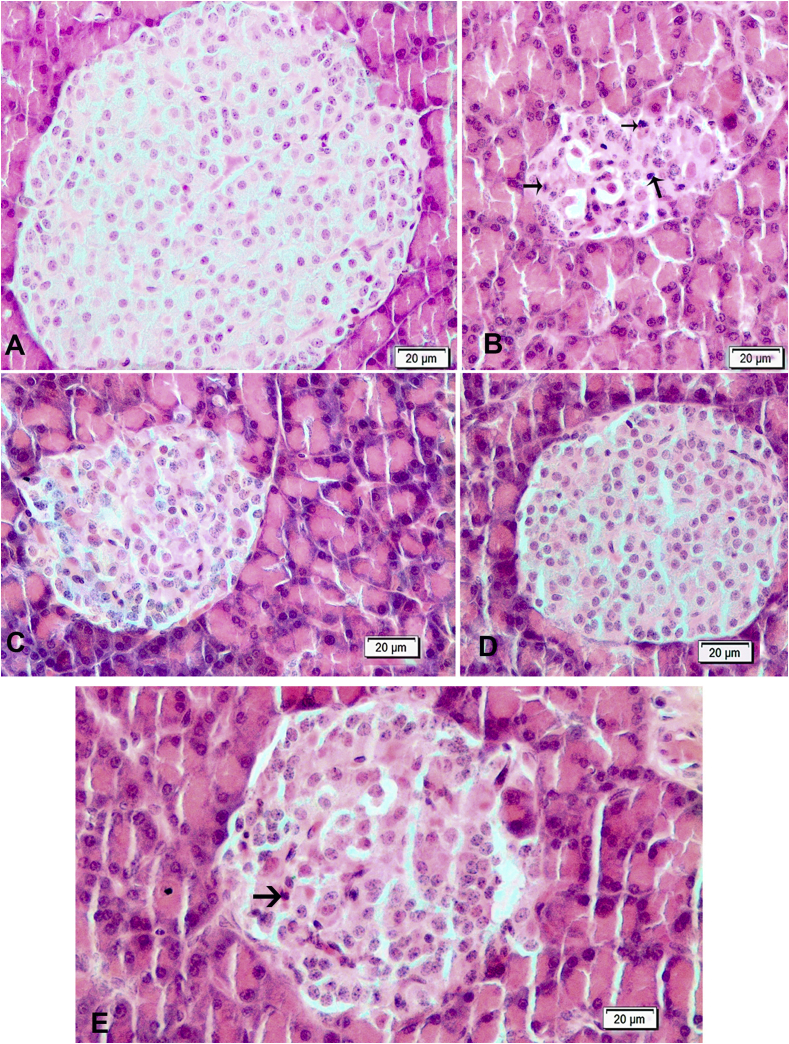
Hematoxylin and eosin-stained sections of pancreas. **A)** Control group: Normal histological appearance of islets of Langerhans. **B)** Diabetic group: Note that islets of Langerhans is atrophic, and there are hydropic degeneration and necrosis (arrows) of some cells of islets of Langerhans. **C)** Diabetic + *L. nobilis* treated group: Almost normal histological appearance of islets of Langerhans. **D)***L. nobilis* group: Normal histological appearance of pancreas. **E)** Diabetic + Acarbose treated group: Showing slight hydropic degeneration and a few necrotic cells of islets of Langerhans. Bar = 20 μm.

Histological views in cells of Langerhans islet in a Control and LN groups were normal (Fig. 2-A and D). The diabetic rats had degenerative and necrotic changes in the cells of Langerhans islet. As a result, atrophied islets which is cells with degenerative and picnotic nucleus had deteriorated and shrunken architecture (Fig. 2-B). Partially, Langerhans islet were preserved in rats in DLN group (Fig. 2-C). A significant recovery in DA group was observed in the islets (Fig. 2-E).

#### 3.3.3 Kidney

**Fig. 3:**
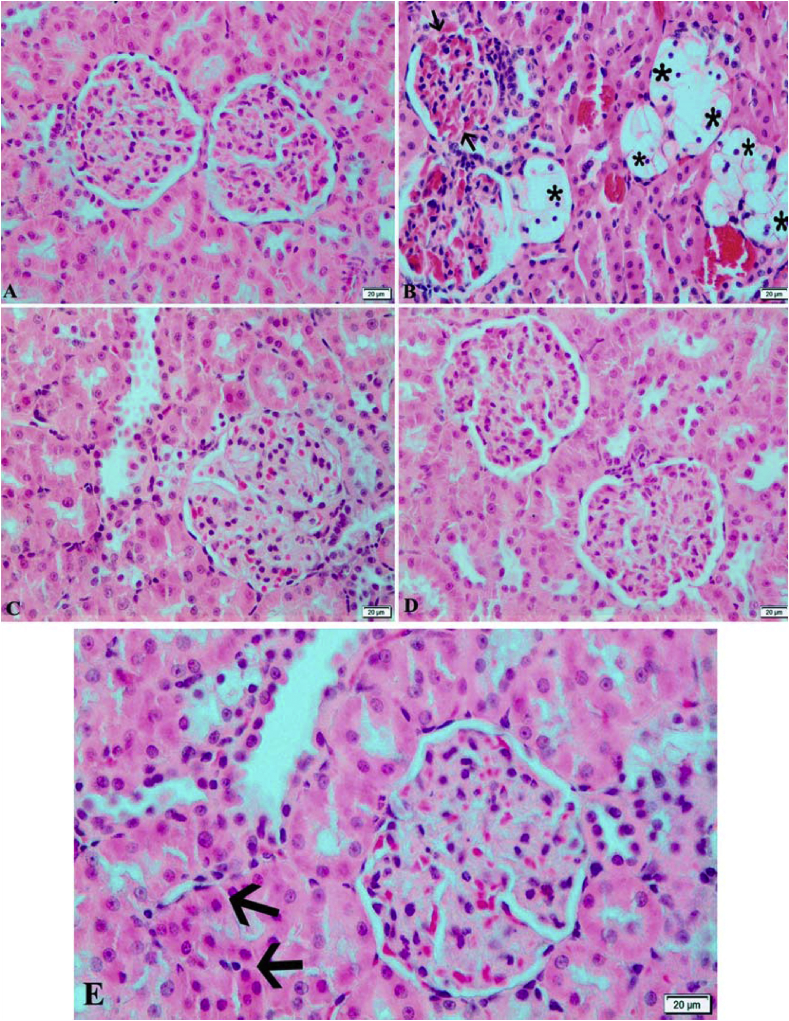
Hematoxylin and eosin-stained sections of kidney. **A)** Control group: Normal histological appearance. **B)** Diabetic group: Showing adhesions (arrows) between the glomerulus and *Bowman* capsule, and severe degeneration (stars) of tubular epithelium. **C)** Diabetic + *L. nobilis* treated group: Almost normal histological appearance of tubular epithelium and glomerulus. **D)***L. nobilis* group: Normal histological appearance of tubular epithelium and glomerulus. **E)** Diabetic + Acarbose treated group: showing degenerative and necrotic changes (arrows) in some tubular epithelium. Bar = 20 μm.

Kidney had normal histological view in both Control and LN groups (Fig. 3-A and D). Severe balloon degeneration and necrosis of tubular epithelium, vascular congestion, degeneration and necrosis of podocytes of glomeruli of kidney of diabetic rats. Inflammatory cells were focally detected in the periglomerular areas. It were adhesion Bowman capsule in some Glomeruli (Fig. 3-B). Slight degeneration and necrosis were determined in some parts of the tubular epithelial cells and dilation of some lymphatic in the kidney of rats in DLN group. Additionally, seldom adhesions in bowman capsule of some glomeruli were observed (Fig. 3-C). DA group were similar to DLN group (Fig. 3-E).

### 3.4 Immunohistochemical evaluation

**Fig. 4:**
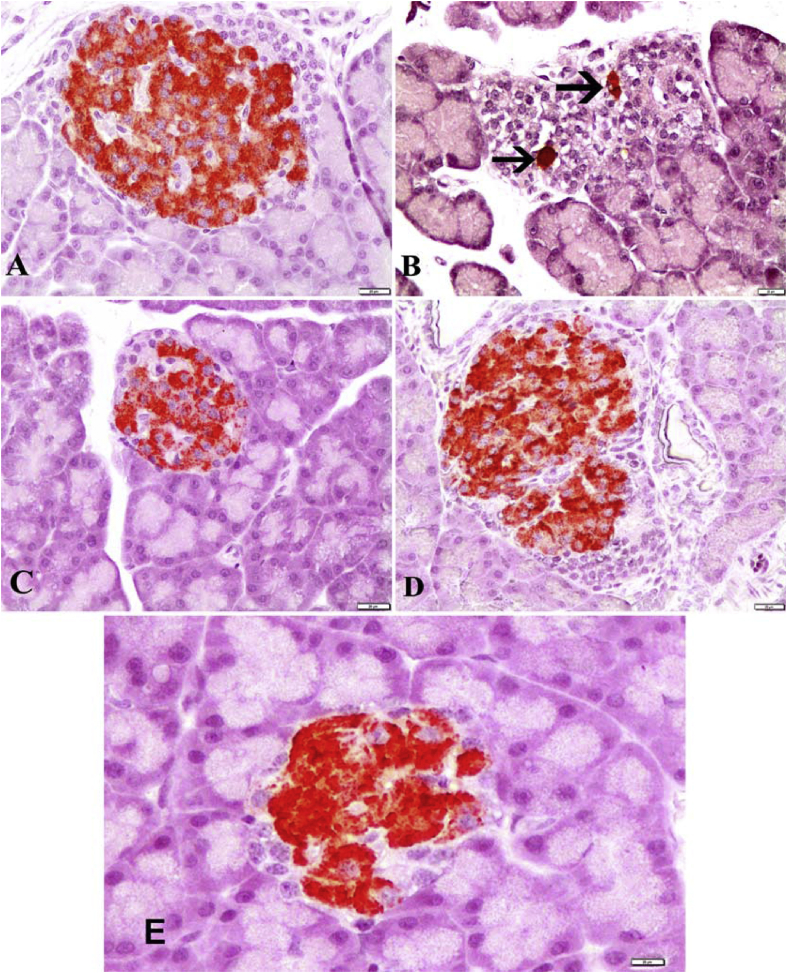
Immunohistochemical staining of islets of Langerhans of pancreas by streptavidin-peroxidase (ABC) method. **A)** Control group: Strong insulin immunoreactivity in β cells, which occupy most of the islet. **B)** Diabetic group: Weak insulin immunoreactivity in a few β cells (arrows). **C)** Diabetic + *L. nobilis* treated group: Moderate insulin immunoreactivity in β cells. **D)***L. nobilis* group: Strong insülin immunoreactivity in β cells. **E)** Diabetic + Acarbose treated group: Moderate insulin immunoreactivity in β cells. Bar = 20 μm.

The insulin secreting β-cells represented the major cell population of the islets, occupying mainly the central zone in the Langerhans islets. Positive insulin expression was seen in the form of dark brown granules present in the cytoplasm of β-cells. Strong insulin immunoreaction were found in the Langerhans islets of Control and LN groups (Fig. 4-A and D). Immunoreactivity in the Diabetes group had dramatically reduced according to the control group and only a few β-cells displayed minimal insulin immunoreaction (Fig. 4-B). In diabetic rats treated *L. nobilis* extracts was increased in the number and percentage area of reactive β-cells an apparent observed, as compared with the diabetic group. Moderate insulin immunoreaction in the DLN group were detected compared to diabetic rats. (Fig. 4-C). DA group showed significantly recovery compared to Diabetes group (Fig. 4-E).

### 3.5 Biochemical results

The glucose concentration decreased significantly in both diabetic rats treated with *L. nobilis* and acarbose (*p* < 0.05) (Table 3). The levels of alanine aminotransferase (ALT) enzyme were significantly (*p* < 0.05) decreased in *L. nobilis* diabetic treated rats compared to diabetic group rats. Also ALT level was significantly (*p* < 0.05) lower in acarbose diabetic treated group as compared to that of diabetes group (Table 3).

**Table 3:** Changes in the serum of Glucose(mg dL ^−1^), ALT(U l ^−1^), AST(U l ^−1^), GGT(U l ^−1^), ALP(U l ^−1^), Urea(mg dL ^−1^), Calcium(mg dL ^−1^), Magnesium(mg dL ^−1^), Phosphor(mg dL ^−1^), Total protein(g dL ^−1^), Albumin(mg dL ^−1^), Creatinine(mg dL ^−1^), and Iron(μmol L ^−1^) levels in different groups under study (mean ± standard error).

BC	G				
	C	D	DLN	DA	LN
ALT	49.16 ± 1.07^a^	251.83 ± 46.91^b^	51.83 ± 10.99^a^	34.16 ± 1.53^a^	77.66 ± 23.41^a^
AST	136.50 ± 7.53^a^	547.33 ± 139.46 ^b^	98.50 ± 6.92^a^	110.33 ± 1.28^a^	216.33 ± 41.20^a^
GGT	0.51 ± .0.14^a^	9.16 ± 2.08 ^b^	2.18 ± 1.16^a^	0.16 ± 0.16^a^	1.08 ± .0.40^a^
ALP	203.16 ± 9.14^a^	678.50 ± 120.73 ^b^	654.33 ± 139.94 ^b^	281.16 ± 25.29^a^	366.50 ± 79.07^a,b^
Urea	31.61 ± 0.91^c^	53.76 ± 1.22 ^d^	19.90 ± 0.90 ^b^	15.05 ± 0.27^a^	30.56 ± 2.69^c^
Ca	11.10 ± 0.12^a^	10.21 ± 0.22^a^	12.58 ± 0.93 ^b^	11.35 ± 0.25^a,b^	10.98 ± 0.30^a^
Mg	3.52 ± 0.08 ^b^	3.50 ± 0.18 ^b^	3.13 ± 0.39^a,b^	2.64 ± 0.04^a^	3.51 ± 0.22 ^b^
P	9.03 ± 0.11 ^b^	9.10 ± 0.45 ^b^	7.39 ± 1.59 ^b^	4.49 ± 0.13^a^	9.75 ± 1.02 ^b^
TP	6.64 ± 0.07^c^	6.23 ± 0.10^a,b^	5.90 ± 0.13^a^	5.87 ± 0.05^a^	6.57 ± 0.21 ^b,c^
ALB	3.65 ± 0.02 ^b^	3.17 ± 0.07^b^	3.56 ± 0.10 ^b^	3.35 ± 0.01^a^	3.58 ± 0.09 ^b^
CK	725.6 ± 85.10 ^b^	664.1 ± 60.77 ^b^	282.1 ± 51.01^a^	331.6 ± 16.01^a^	809.6 ± 52.38 ^b^
Fe	32.08 ± 1.57 ^b^	17.20 ± 3.55^a^	32.20 ± 6.95 ^b^	35.15 ± 3.60 ^b^	40.30 ± 5.60 ^b^

*BC; biochemical tests were ALT (alanine aminotransferase), AST (aspartate aminotransferase), GGT (gamma-glutamyltransferase), ALP (alkaline phosphatase), Ca (calcium), Mg (magnesium), P (phosphor), TP (total protein), ALB (albumin), CK (creatinine kinase), Fe (iron).

*G; groups were C (control group), D (diabetic group), DLN (diabetic with *Laurus nobilis* trated group), DA (diabetic with drug treated group), LN (*Laurus nobilis* fed group).

*a - c Means with the same column are not significantly different *P* ˃ 0.05. *^a-c^ Means with different column are statistically significant *P* ˂ 0.05.

The mean values of aspartate aminotransferase (AST) activities were significantly decreased in both diabetic rats treated with *L. nobilis* and acarbose (*p* < 0.05), the level of AST was lower in *L. nobilis* treated rats as comparison other rat groups (Table 3). As shown in (Table 3), the mean values of Gamma-glutamyltransferase (GGT) activities were significantly decreased in all groups when compared with diabetes group (*p* < 0.05). However, the differences within other groups were not significant (*p* ˃ 0.05).

A statistically non-significant decrease (*p* > 0.05) found in alkaline phosphatase (ALP) level *L. nobilis* diabetes treatment group in comparison with diabetes groups. In addition, statistically significant decrease (*p* < 0.05) can be seen in both diabetes drug treatment acarbose and undiabetes treatment of *L. nobilis* extracts as compared with diabetes group (Table 3).

The mean values of Urea level were significantly decreased in DLN extracts group as compared with diabetic groups (*p* < 0.05). Diabetic treated group of rats showed a significant high level of calcium as compared to other group of rats (*p* < 0.05). The *L. nobilis*-fed group was no significant when compared with control group (Table 3). No significant difference in magnesium level was observed in treated rats group with the diabetic group (*p* > 0.05), (Table 3). In the phosphor (*p*) level did not show any significant difference in *L. nobilis* extract compared to both diabetic and control group of rats (*p* > 0.05) (Table 3).

Statistically non-significant and slight reduction found in the total protein (TP) levels as compared with diabetic group. *L. nobilis* leave extract and control group are relative and significant when compared with diabetic group (*p* > 0.05), (Table 3).

A insignificantly (*p* > 0.05) increased can be seen in the Albumin (ALB) level in the *L. nobilis* leave extract group in comparison with diabetic group (Table 3). The level of creatinine kinase (CK) was decreased significantly (*p* < 0.05) in the diabetic *L. nobilis* group in comparison with the diabetic group (Table 3). Iron (Fe) levels in the diabetic rats were treated with *L. nobilis* significantly increased when compared to diabetic group rats, there was no significant difference in the treated diabetes groups with control group in the levels of Fe (*p* > 0.05), (Table 3).

## 4 Discussion and conclusion

Conventional therapies for DM have many side effects and high rate of secondary failure. On the other hand herbal extracts are expected to have similar efficacy with fewer side effects than conventional drugs [33]. Nowadays, more than 1200 plant species are used to treat symptoms of DM, the hypoglycemic property of almost 50% of these traditionally consumed medicines has been experimentally tested [34].

In our study detected weight loss in all STZ-induced rats group when comparisons with control group, and exhibited hyperglycaemia in STZ-induced rats with decrease in serum glucose levels in the treated diabetic groups. *L. nobilis* leaves extracts (LNLE) inhibit the development of diabetes induced by STZ and decrease serum glucose levels. LNLE treatment did not induce a significant change in the body weight of the diabetic rats, however, LNLE had a significant decrease in the blood glucose levels for 28 days of the diabetic rats’ treatment group.

In this study, we observed histopathological and biochemical changes in STZ-induced rats and these changes were reduced with LNLE treatment. The promising mechanism by which LNLE mediated its antidiabetic effect could be by potentiation of pancreatic secretion of insulin from existing β-cells of islets, as was evident by the significant decrease in the level of glucose in the extract treated animals.

The hypoglycemic activity of LNLE was compared with acarbose, a standard hypoglycemic drug, since the results of the present study, it may be suggested that the mechanism of action of *L. nobilis* may be similar to acarbose action. In this essay indicated that the pathological effects in rats liver and pancreas tissues induced by STZ were reversible and normalized by received extracts bay leaves for 4 weeks of experiment, this could be attributed to its antidiabetic effects. Several studies have reported that the hepatocytes of STZ-induced diabetic rats showed cytoplasmic alterations, sinusoidal dilation and congestion, periportal inflammation, showed kupffer cells activation, cytoplasmic vacuolization of hepatocytes and necrosis [35].

The hepatocyte nuclei were generally enlarged and sometimes showed irregular contours and intranuclear inclusions [36]. In pancreatic sections of untreated diabetic rats disclosed that the islets were comparatively small and shrunken and severe degenerative changes in the pancreatic islets, mainly at the center of the islets and karyolysis of the nuclei was visible [37]. The *L. nobilis* treated diabetic rats were reversed and the normalization of pancreatic architecture revealed vacuolations of β-cells was observed.

In addition regarding the immunohistochemistry, different sizes of islets of pancreas were observed with increasing immunoreaction to insulin antibody in β-cells treated with *L. nobilis* leaves extracts comparison to diabetic group, langerhans with increasing insulin immunoreactivity in cytoplasm of its β-cells, in the number and percentage area of reactive β-cells, areas of dark brown staining (strong positive) for insulin antibody were seen in islet β-cells cytoplasm from diabetic treated by *L. nobilis*.

In STZ-induced diabetic rats the liver was necrotized. An increase in the activities of ALT, AST and GGT in plasma might be mainly an indication of the hepatotoxic effect of STZ [38]. ALT, AST and GGT were significantly decreased in diabetic bay leaves treatment, and related to normal control group. In contrast diabetic group significantly higher level of enzymatic liver function test observed [39]. Al Chalabi et al. [40] reported that the bay leave extract resulted in a decrease in fasting blood glucose and a higher level of fasting insulin, also LDL, ALT, and AST were also decreased, whereas HDL and body weight increased in groups of diabetic rats relative to control not treated groups. In another study recommended that the *L. nobilis* tea consumption in healthy volunteers can improve blood lipid profile (HDL level increased and a small decrease in levels of LDL and triglycerides) and this implies a possible positive impact on the risk reduction of coronary heart disease [41]. Similar results were reported by Casamassima et al. [42] and investigated a substantial reduction in blood lipid profile, glycemic profile and liver enzymes, with decreased levels of LDL, ALT and AST, and increased HDL, has resulted from dietary incorporation of dried bay leaves meal.

Treatment of the diabetic rats with LNLE reduced the activity of these enzymes in plasma compared to the diabetic untreated group and consequently alleviated liver damage caused by STZ-induced diabetes and indicated the hepato protective role in preventing diabetic complications. The diabetic rats treated with bay leave extract displayed a statistically significant reduction in the urea and creatinine levels with respect to diabetic rats. In this sense, kidney failure is manifested by increasing urea and creatinine but a decrease indicates clinical improvement [43].

There's still clearly a significant dispute about the use of natural or cultivated plants, which has both positive and negative aspects in biophysical terms, as well as in terms of economics. *L. nobilis* is a significant socioeconomic evergreen tree belonging to the *Lauraceae* family. *L. nobilis* are used as antihyperglycaemic herbs, used to treat bacterial and fungal contaminations, to treat eructation, flatulence and gastrointestinal problems. It also exhibits anti-inflammatory, anticonvulsive, antiepileptic and antioxidant properties [44,45].

In conclusion, from this study, based on the experimental findings, it was suggested that administration of *L. nobilis* leave extracts, at a safe dose level, significantly suppressed STZ-induced diabetic rats. We believe that further preclinical research into the utility of *L. nobilis* treatment may indicate its suitability as a potential treatment in diabetic patients, our results expressed that leave extracts of *L. nobilis* has valuable effect on blood glucose level and ameliorative effect on regeneration of pancreatic islets. It also restored the altered liver enzymes (ALT, AST, and GGT), urea, creatine kinase, total protein levels calcium and Fe to near normal. It may be used as a therapeutic agent in the management of diabetes mellitus.

## Author contributions

All authors contribute to this research study.

Rebin R. Mohammed, Abdullah K. Omer, Avin K, Ahmed: Data collection and analysis, original draft preparation, Zabit Yener, Ahmet Uyar: Study design, conceptualization and writing, review and editing.

## 5 Provenance and peer review

Not commissioned, externally peer reviewed.

## Funding

The authors wish to acknowledge 10.13039/501100010760Van Yuzȕncu Yıl University for the support (Grant number 2015-SBE-YL309).

## Ethical approval

All experimental protocols were approved by the Experimental Animal Center of Van Yȕzȕncȕ Yıl University, Turkey.

## Consent

All experimental protocols were approved by the Experimental Animal Center of Van Yȕzȕncȕ Yıl University, Turkey.

## Guarantor

Rebin R.Mohammed (on behalf of all authors).

## Declaration of competing interest

There is no conflict of interest.
