# Supplementary material for: Biomedical effects of Laurus nobilis L. leaf extract on vital organs in streptozotocin-induced diabetic rats: Experimental research
Source: Ann Med Surg (Lond). 2020 Nov 21;61:188–97. doi: 10.1016/j.amsu.2020.11.051 (PMC7817776; doi:10.1016/j.amsu.2020.11.051)
Supplement: Multimedia component 1 [file mmc1.doc]

**The ARRIVE Guidelines**

**Animal Research: Reporting *In Vivo* Experiments**

|  | **Item** | **Recommendation** |
| --- | --- | --- |
| **TITLE** | **1** | **Biomedical effects of *Laurus nobilis L*. leaf extract on vital organs in streptozotocin-induced diabetic rats: Experimental research** |
| **ABSTRACT** | **2** | **Diabetes mellitus (DM) has been treated with herbs for centuries and many herbs reported to exert antidiabetic activity. *Laurus nobilis* is an aromatic herb belonging to the *Lauraceae* family, commonly known as bay. This study aimed to investigate the activity of *Laurus nobilis* leave extracts on histopathological and biochemical changes in β-cells of streptozotocin (STZ)-induced diabetic rats. Thirty healthy adult male albino rats were included in the study and divided equally into 5 groups for 4 weeks, control group (C), diabetic group (D), diabetic *Laurus nobilis* extract group (DLN), *Laurus nobilis* extract group (LN) and diabetic acarbose (DA) group. The results revealed that the administration of LN to diabetic rats significantly decreased (*p* < 0.05) blood glucose within 4 weeks of treatment. Additionally, LN also showed protection of liver and kidney functions. The glucose concentration decreased significantly in both diabetic rats treated with *L. nobilis* and acarbose (*p* < 0.05), the levels of aspartate aminotransferase (AST), gamma-glutamyltransferase (GGT) and alanine aminotransferase (ALT) enzyme were insignificantly decreased in both diabetic rats treated with *L. nobilis* and acarbose (*p* ˃ 0.05). Outcomes of this study said that leave extracts of *L. nobilis* has valuable effect on blood glucose level and ameliorative effect on regeneration of pancreatic islets, it also restored the altered liver enzymes, urea, creatine kinase, total protein levels, calcium and ferritin to near normal.**  **Keywords: Diabetes mellitus, *Laurus nobilis*, Streptozotocin, Histopathology, Blood glucose.** |
| **INTRODUCTION** |  |  |
| - **Background** | **3** | 1. **This study aimed to investigate the activity of *Laurus nobilis* leave extracts on histopathological and biochemical changes in β-cells of streptozotocin (STZ)-induced diabetic rats.** 2. **An** **adult male albino rats were included in the study.** |
| - **Objectives** | **5** | **Outcomes of this study said that leave extracts of *L. nobilis* has valuable effect on blood glucose level and ameliorative effect on regeneration of pancreatic islets, it also restored the altered liver enzymes, urea, creatine kinase, total protein levels, calcium and ferritin to near normal.** |
| **METHODS** |  |  |
| - **Ethical statement** | **5** | **All experimental protocols were approved by the Experimental Animal Center of Van Yuzuncu Yil University, Turkey.** |
| - **Study design** | **6** | **Thirty healthy adult male albino rats were included in the study and divided equally into 5 groups for 4 weeks, control group (C), diabetic group (D), diabetic *Laurus nobilis* extract group (DLN), *Laurus nobilis* extract group (LN) and diabetic acarbose (DA) group.** |
| - **Experimental procedures** | **7** | **In the present study, thirty male Wistar rats, weighting about 160–200 g with averagely 7 weeks old were randomly divided into five groups based on treatment each group containing 6 animals. All animals were housed under safe laboratory conditions in a temperature-controlled room (22–24 ̊C) and kept on a 12 h light/dark cycle. Blood glucose and body weight were monitored before treatment once a week throughout 4 weeks of experimental period.** |
| - **Experimental animals** | **8** | **Thirty male Wistar rats, weighting about 160–200 g with averagely 7 weeks old.** |
| - **Housing and husbandry** | **9** | **Van Yűzuncű Yil Universityies Animal House.** |
| - **Sample size** | **10** | **Male Wistar rats, weighting about 160–200 g with averagely 7 weeks old.** |
| - **Allocating animals to experimental groups** | **11** | **30 male Wistar rats, weighting about 160–200 g with averagely 7 weeks old were randomly divided into 5 groups based on treatment each group containing 6 animals.** |
| - **Experimental outcomes** | **12** | **Outcomes of this study said that leave extracts of *L. nobilis* has valuable effect on blood glucose level and ameliorative effect on regeneration of pancreatic islets, it also restored the altered liver enzymes, urea, creatine kinase, total protein levels, calcium and ferritin to near normal.** |
| - **Statistical methods** | **13** | **Statistical analyses were performed by using ‘IBM SPSS Alan C. Elliott software. ANOVA test was applied to analysis the significant differences in all groups.** |
| **RESULTS** |  |  |
| - **Baseline data** | **14** | **In our study detected weight loss in all STZ-induced rats group when comparisons with control group, and exhibited hyperglycaemia in STZ-induced rats with decrease in serum glucose levels in the treated diabetic groups. *L. nobilis* leaves extracts (LNLE) inhibit the development of diabetes induced by STZ and decrease serum glucose levels. LNLE treatment did not induce a significant change in the body weight of the diabetic rats, however, LNLE had a significant decrease in the blood glucose levels for 28 days of the diabetic rats’ treatment group.** |
| - **Numbers analyzed** | **15** | **Experimental animals were randomly divided into 5 groups; each group was included 6 animals. The examination period was continuous for four weeks.** |
| - **Outcomes and estimation** | **16** | **from this study, based on the experimental findings, it was suggested that administration of *L. nobilis* leave extracts, at a safe dose level, significantly suppressed STZ-induced diabetic rats. We believe that further preclinical research into the utility of *L. nobilis* treatment may indicate its suitability as a potential treatment in diabetic patients, our results expressed that leave extracts of *L. nobilis* has valuable effect on blood glucose level and ameliorative effect on regeneration of pancreatic islets. The bioactive components in bay leaves have been shown to have effects on insulin sensitivity, glucose uptake, antioxidant status, inflammatory response, and glucose emptying. It also restored the altered liver enzymes (ALT, AST, and GGT), urea, creatine kinase, total protein levels calcium and Fe to near normal. It may be used as a therapeutic agent in the management of diabetes mellitus.** |
| - **Adverse events** | **17** | **Not seen** |
| **DISCUSSION** |  |  |
| - **Interpretation/scientific implications** | **18** | **Outcomes of this study said that leave extracts of *L. nobilis* has valuable effect on blood glucose level and ameliorative effect on regeneration of pancreatic islets, it also restored the altered liver enzymes, urea, creatine kinase, total protein levels, calcium and ferritin to near normal.** |
| - **Generalisability/translation** | **19** | **The bioactive components in bay leaves have been shown to have effects on insulin sensitivity, glucose uptake, antioxidant status, inflammatory response, and glucose emptying. It also restored the altered liver enzymes (ALT, AST, and GGT), urea, creatine kinase, total protein levels calcium and Fe to near normal. It may be used as a therapeutic agent in the management of diabetes mellitus.** |
| - **Funding** | **20** | **Van Yȕzȕncȕ Yıl University.** |
